# Influence of sodium thiosulfate on coronary artery calcification of patients on dialysis: a meta-analysis

**DOI:** 10.1080/0886022X.2023.2254569

**Published:** 2023-09-27

**Authors:** Chong Huang, Zhibing Duan, Chengyun Xu, Yan Chen

**Affiliations:** Department of Nephrology, the Second Affiliated Hospital of Nanchang University, Nanchang, China

**Keywords:** Dialysis, coronary artery calcification, sodium thiosulfate, efficacy, meta-analysis

## Abstract

Coronary artery calcification (CAC) is common in dialysis patients and is associated with a higher risk of future cardiovascular events. Sodium thiosulfate (STS) is effective for calciphylaxis in dialysis patients; however, the influence of STS on CAC in dialysis patients remains unclear. This systematic review and meta-analysis were conducted to evaluate the effects of STS on CAC in patients undergoing dialysis. PubMed, Embase, Cochrane Library, CNKI, and Wanfang databases were searched from inception to 22 March 2023 for controlled studies comparing the influence of STS versus usual care without STS on CAC scores in dialysis patients. A random effects model incorporating the potential influence of heterogeneity was used to pool the results. Nine studies, including two non-randomized studies and seven randomized controlled trials, were included in the meta-analysis. Among these, 365 patients on dialysis were included in the study. Compared with usual care without STS, intravenous STS for 3–6 months was associated with significantly reduced CAC scores (mean difference [MD] = −180.17, 95% confidence interval [CI]: −276.64 to −83.70, *p* < 0.001, I^2^ = 0%). Sensitivity analysis limited to studies of patients on hemodialysis showed similar results (MD: −167.33, 95% CI: −266.57 to −68.09, *p* = 0.001; I^2^ = 0%). Subgroup analyses according to study design, sample size, mean age, sex, dialysis vintage of the patients, and treatment duration of STS also showed consistent results (p for subgroup differences all > 0.05). In conclusion, intravenous STS may be effective in attenuating CAC in dialysis patients.

## Introduction

Vascular calcification, which involves coronary artery calcification (CAC), is a common morbidity in patients with end-stage renal disease undergoing dialysis [1,2]. In a recent Chinese nationwide cohort study of 1489 patients undergoing maintenance dialysis, progression of CAC developed in 69.6% of patients during a 4-year follow-up duration [3]. Moreover, CAC progression has been independently associated with a higher risk of all-cause mortality and an increased incidence of major adverse cardiovascular events (MACEs) in these patients [3]. Similar results have been reported by studies conducted in other countries. An early cohort study in the United States including 1541 patients with chronic kidney disease (CKD) showed that one standard deviation of log higher CAC score was significantly associated with a 40% higher risk of cardiovascular disease, 44% higher risk of myocardial infarction, and 39% higher risk of heart failure after adjusting for important risk factors [4]. Moreover, it has been also shown in a previous cross-sectional study that no significant difference was observed in the severity and progression of CAC between patients with peritoneal dialysis and hemodialysis [5]. The pathogenesis of CAC in patients on dialysis is complicated [6], and aging has been recognized as a major risk factor [7,8]. In addition, the current evidence does not seem to support the notion that the risk of CAC differs among patients undergoing hemodialysis or peritoneal dialysis [5,9]. Although various interventions have been proposed to reduce CAC progression in patients with CKD, evidence-based therapies remain limited [10].

Sodium thiosulfate (STS) has been suggested to be effective for calciphylaxis in dialysis patients, although the results of previous studies have been inconsistent [11,12]. Several studies have demonstrated that STS has vasodilatory and antioxidant effects and can chelate calcium deposits to form soluble calcium thiosulfate complexes, suggesting that STS may also be effective in attenuating the progression of CAC [13–16]. However, clinical studies evaluating the influence of intravenous STS on CAC progression in dialysis patients are still limited, generally with small sample sizes and inconsistent results [17–25]. Therefore, this systematic review and meta-analysis were performed to comprehensively evaluate the effect of STS on CAC progression in patients on dialysis.

## Methods

This study was designed and implemented according to the Cochrane Handbook guidelines [26] and the PRISMA (Preferred Reporting Items for Systematic Reviews and Meta-Analyses) statement [27,28]. The PRISMA Checklist of the meta-analysis is shown in Supplementary File 1.

### Search strategy

A combination of strategies was used to search PubMed, Embase, Cochrane Library, China National Knowledge Infrastructure (CNKI), and Wanfang databases for relevant studies with: (1) "thiosulfate" OR "thiosulfates" OR "disodium salt" OR "hyposulfite" OR "thiosulfate" OR "sodothiol" OR "sulfactol" OR "sulfothiorine"; (2) "calcification" OR "coronary"; and (3) "dialysis" OR "hemodialysis.” The detailed search terms used for each database are shown in Supplementary File 2. Relevant clinical studies have been limited to humans. We also manually searched for reference lists to review and original articles that were related to the topic. Database searches were conducted on 22 March 2023.

### Study selection

Studies were included if they fulfilled the following criteria according to the PICOS principles.

P (patients): adult patients on hemodialysis or peritoneal dialysis, without the limitations of dialysis vintage.

I (intervention): intravenous STS on the basis of usual care.

C (control): usual care only.

O (outcome): difference in CAC score changes between patients allocated to the intervention and control groups. The evaluation of CAC and quantification of CAC scores were in accordance with the methods used in the included studies.

S (study design): controlled clinical studies, including non-randomized controlled studies (NRCTs) and randomized controlled trials (RCTs), published as full-length articles in peer-reviewed journals.

Studies that did not include patients on dialysis, those investigating the combined effect of STS and other interventions, single-arm studies without controls, or studies not evaluating the outcome of CAC were excluded. For studies with overlapping patients, the study with the largest sample size was included.

### Data extraction and quality assessment

Data extraction, mining, and quality evaluations were performed by two independent authors (CH and ZD). The agreement between the two authors was determined by using Cohen’s Kappa value. If a disagreement occurred during the systematic search, data extraction, and study quality evaluation, the corresponding author was consulted to resolve this inconsistency. Information regarding publication details (first author, publication year, and study country), study design (NRCTs or RCTs), patient characteristics (diagnosis, demographic information, and dialysis vintage), intervention (dosage and duration of STS treatment), and methods for quantification of CAC were extracted. For NRCTs, the quality of the study was evaluated using the Risk of Bias in Non-randomized Studies of Interventions (ROBINS-I), which uses the Cochrane-approved risk of bias approach and focuses on internal validity, assessing bias in seven domains [29]. We evaluated the quality of RCTs using the Cochrane Risk of Bias Tool [26] in accordance with the following criteria: (1) random generation of sequences; (2) concealing allocations; (3) blinding of participants and staff; (4) blinding of outcome assessors; (5) presenting incomplete outcome data; (6) reporting selective results; and (7) other potential biases.

### Statistical analysis

The influence of STS on CAC scores of dialysis patients compared to usual care was summarized as mean difference (MD) and corresponding 95% confidence intervals (CIs). The Cochrane Q test was performed [30]. Heterogeneity was also estimated by calculating I^2^, and I^2^ >50% suggested significant heterogeneity [31]. A random-effects model was used in the pooled analyses to account for potential heterogeneity and provide a more general conclusion [26]. We conducted a sensitivity analysis to assess whether each study contributed to the pooled meta-analysis results irrespective of whether it was included or excluded [26]. In addition, the sensitivity analysis was limited to patients receiving hemodialysis. A subgroup analysis was also conducted to assess the influence of predefined study characteristics on the outcome, including study design, sample size, mean age, sex, dialysis vintage of the patients, and STS treatment duration. Funnel plots of the standard error of mean difference (SE[MD]) by mean difference (MD) were constructed for assessment of publication bias. In addition, Egger’s regression asymmetry test was conducted to determine publication bias [32]. Statistical significance was defined as *p* < 0.05. Stata software (version 12.0; Stata Corporation) and RevMan (version 5.1; Cochrane, Oxford, UK) were used for statistical analysis.

## Results

### Search results

**Figure 1.**
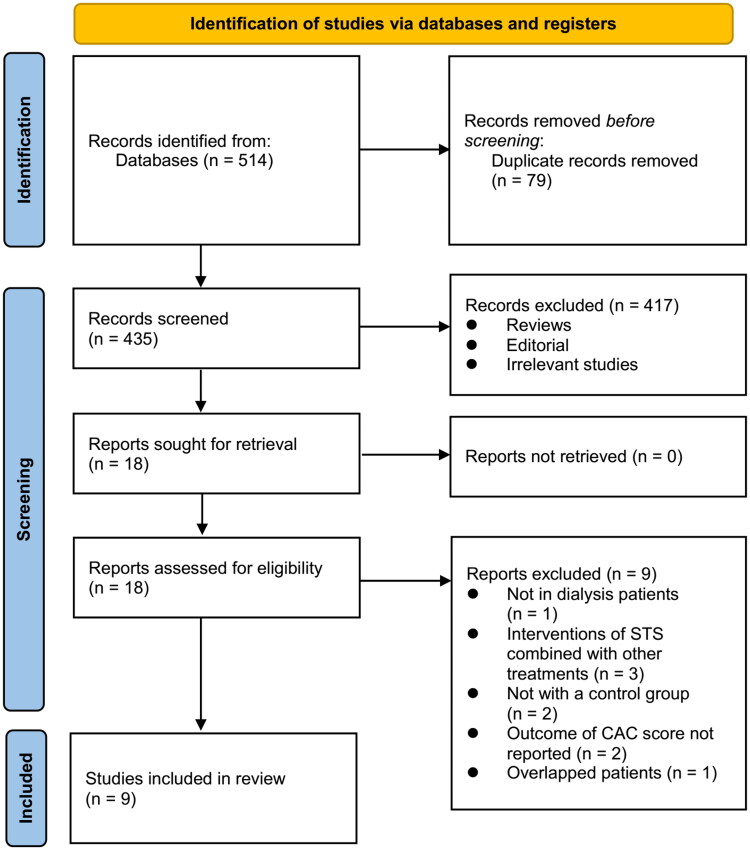
Flowchart of literature search.

A diagram showing how to search the databases and identify studies is shown in Figure 1. By searching the databases, 514 articles were obtained, and 435 were identified after excluding duplicates. Based on the title and abstract, 417 articles were subsequently excluded, mainly because their objectives were irrelevant. Full-text review was performed on 18 articles, and nine of them were further excluded for the reasons illustrated in Figure 1. The final analysis included nine studies [17–25] in total. The agreement between reviewers in study selection reached a kappa value of 0.90.

### Study characteristics and data quality

An overview of the included studies is presented in Table 1. Overall, nine studies, including two NRCTs [17,18] and seven RCTs [19–25] were included in the meta-analysis. These studies were published between 2010 and 2022 and were conducted in Thailand [17,–20], Bulgaria [18], and China [19,21–25]. Eight of the studies included patients undergoing hemodialysis [17–20, 22–25], and the other study included patients undergoing peritoneal dialysis [21]. Among these, 365 patients on dialysis were included in the study. The sample sizes of the included studies were generally small, varying between 12 and 70. The mean age of the patients was 46–61 years and the proportion of men was 47–60%. The mean dialysis vintage of the patients ranged from 23 to 84 months. A total of 184 patients were allocated to the intravenous STS intervention group based on usual care, and 181 patients were allocated to the control group of usual care only. The doses of intravenous STS were 5.6 g in one study [18], 12.5 g in three studies [17,20,21], and 0.18 k/kg body weight in the other five studies [19,22–25], administered twice or three times per week during or after dialysis. The treatment duration was 3 months in three studies [19,21,22], four months in one study [17], and six months in the other five studies [18,20,23–25]. The Agatston method was used to quantify CAC on computed tomography angiography (CTA) [33] in all included studies. According to Table 2, the quality of each included study was assessed (Table 2). The agreement between reviewers in study data extraction reached a kappa value of 0.85. Two of the NRCTs [17,18] were judged to have a moderate risk of bias according to the domains of ROBINS-I. The detailed quality assessments for the seven RCTs according to the Cochrane Risk of Bias Tool are shown in Table 2. These RCTs were all open-label, and only two of them reported the method of random sequence generation [21,24]. Details of the allocation concealment were not reported in any of the included RCTs.

**Table 1.** Characteristics of the included studies.

Study	Country	Design	Patient diagnosis	No of patients	Mean age (years)	Men (%)	Dialysis vintage (months)	Intervention	Control	Treatment duration (months)	Methods for measuring CAC
Adirekkia 2010 [17]	Thailand	NRCT	Stable chronic hemodialysis patients with a CAC score ≥ 300	32	60	59.4	50	STS 12.5 g iv Biw, over 15–20 min after dialysis	Usual care	4	Agatston method
Yonova 2014 [18]	Bulgaria	NRCT	Hemodialysis patients	12	NR	NR	NR	STS 5.6 g iv Biw, at first 15 min of dialysis	Usual care	6	Agatston method
Yu 2016 [19]	China	RCT	Stable chronic hemodialysis patients with a CAC score ≥ 50	25	46.9	55.6	84	STS 0.18 g/kg iv Tiw, over 30 min after dialysis	Usual care	3	Agatston method
Saengpanit 2018 [20]	Thailand	RCT	Hemodialysis patients with atherosclerosis	50	52.5	56	62	STS 12.5 g iv Biw, during the last hour of hemolysis	Usual care	6	Agatston method
Zhu 2019 [22]	China	RCT	Hemodialysis patients with CAC ≥ 0	46	48.9	47.8	52	STS 0.18 g/kg iv Tiw, over 30 min after dialysis	Usual care	3	Agatston method
Mao 2019 [21]	China	RCT	Peritoneal dialysis patients with CAC ≥ 0	30	56.9	53.3	23	STS 12.5 g iv Biw, over 1h after dialysis	Usual care	3	Agatston method
Zhu 2020 [23]	China	RCT	Hemodialysis patients with atherosclerosis	50	61	48	41	STS 0.18 g/kg iv Tiw, over 30 min after dialysis	Usual care	6	Agatston method
Li 2021 [24]	China	RCT	Stable chronic hemodialysis patients with a CAC score ≥ 0	70	49.2	58.6	40	STS 0.18 g/kg iv Tiw, over 30 min after dialysis	Usual care	6	Agatston method
Bian 2022 [25]	China	RCT	Hemodialysis patients	50	52.3	48	30	STS 0.18 g/kg iv Tiw, over 30 min after dialysis	Usual care	6	Agatston method

CAC: coronary artery calcification; NRCT: non-randomized controlled trials; RCT: randomized controlled trials; NR: not reported; STS: sodium thiosulfate; Biw: twice a week; Tiw: three times a week.

**Table 2.** Study quality evaluation of NRCTs with ROBINS-I and RCTs *via* Cochrane risk of bias Tool.

NRCTs							
Studies	Confounding	Selection of participants	Classification of interventions	Deviations from intended interventions	Missing data	Measurement of outcomes	Selection of reported results
Adirekkia 2010 [17]	Low risk	Moderate risk	Low risk	Low risk	Low risk	Moderate risk	Low risk
Yonova 2014 [18]	Moderate risk	Moderate risk	Low risk	Low risk	Low risk	Low risk	Low risk
RCTs							
Studies	Random sequence generation	Allocation concealment	Blinding in performance	Blinding in outcome detection	Incomplete outcome data	Reporting bias	Other bias
Yu 2016 [19]	Unclear	Unclear	Unclear	Unclear	Low risk	Low risk	Low risk
Saengpanit 2018 [20]	Unclear	Unclear	High risk	High risk	Low risk	Low risk	Low risk
Zhu 2019 [22]	Unclear	Unclear	Unclear	Unclear	Low risk	Low risk	Low risk
Mao 2019 [21]	Low risk	Unclear	High risk	High risk	Low risk	Low risk	Low risk
Zhu 2020 [23]	Unclear	Unclear	High risk	High risk	Low risk	Low risk	Low risk
Li 2021 [24]	Low risk	Unclear	High risk	High risk	Low risk	Low risk	Low risk
Bian 2022 [25]	Unclear	Unclear	Unclear	Unclear	Low risk	Low risk	Low risk

NRCTs: non-randomized controlled trials; RCTs: randomized controlled trials; ROBINS-I: risk of bias in non-randomized Studies - of Interventions.

## Meta-analysis results

**Figure 2.**
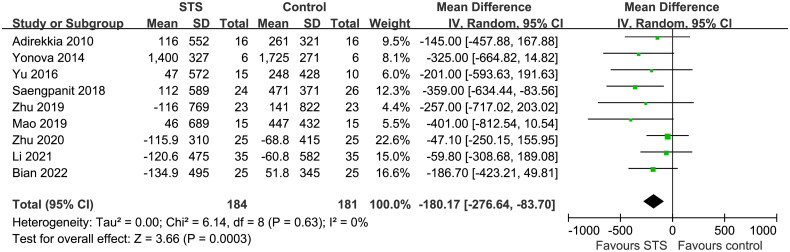
Forest plots for the meta-analysis of the influence of intravenous STS on CAC scores in dialysis patients.

**Figure 3.**
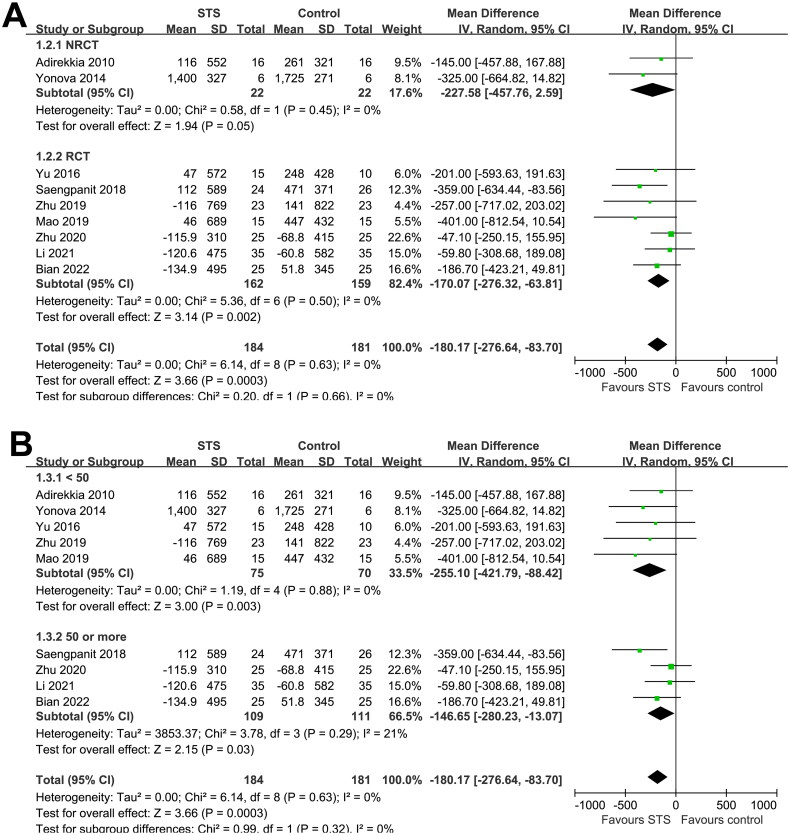
Forest plots for the subgroup analyses of the influence of intravenous STS on CAC scores in dialysis patients according to study design and sample size; A, subgroup analysis according to study design; and B, subgroup analysis according to sample size.

**Figure 4.**
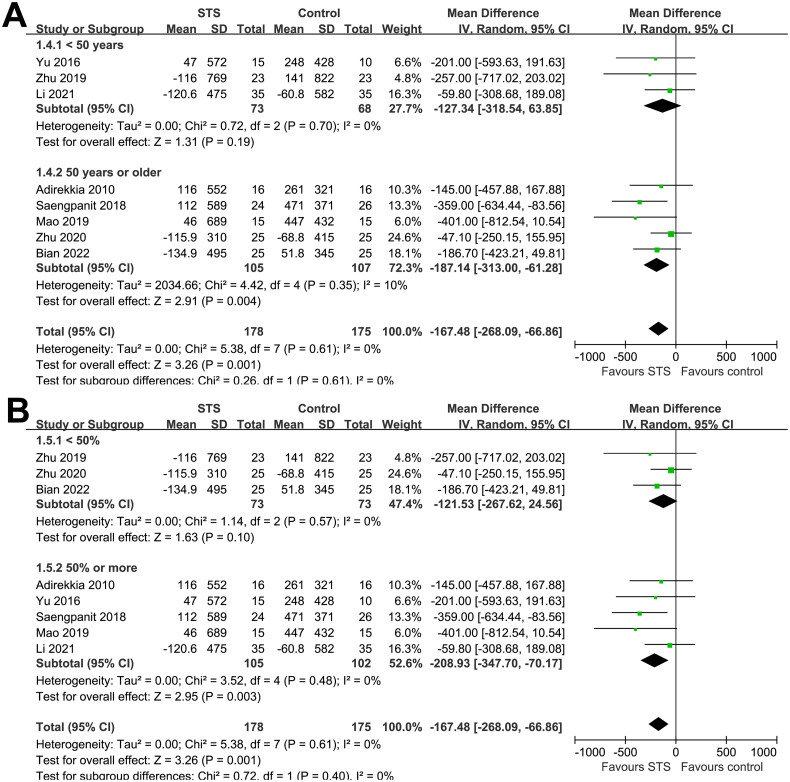
Forest plots for the subgroup analyses of the influence of intravenous STS on CAC scores in dialysis patients according to the mean age and proportion of men in each study; A, subgroup analysis according to mean age of the patients; and B, subgroup analysis according to Proportions of men.

**Figure 5.**
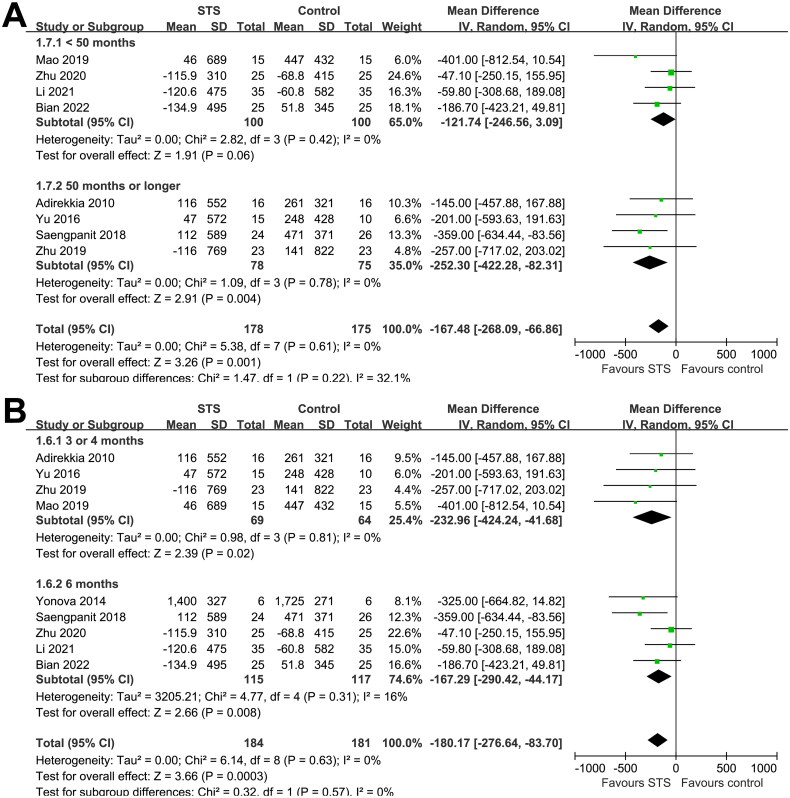
Forest plots for the subgroup analyses of the influence of intravenous STS on CAC scores in dialysis patients according to the dialysis vintage and STS treatment duration; A, subgroup analysis according to the dialysis vintage; and B, subgroup analysis according to STS treatment duration.

Overall, the pooled results of nine studies showed that, compared to usual care without STS, intravenous STS for 3–6 months was associated with significantly reduced CAC scores (MD = −180.17, 95% CI: −276.64 to −83.70, *p* < 0.001; Figure 2) without significant heterogeneity (p for Cochrane Q test = 0.63, I^2^ = 0%). Sensitivity analysis excluding one study at a time showed consistent results (MD: −155.16, −218.97, *p* < 0.05). In particular, the sensitivity analysis limited to studies of patients on hemodialysis showed similar results (MD, −167.33; 95% CI: −266.57 to −68.09, *p* = 0.001; I^2^ = 0%). Moreover, subgroup analyses showed consistent results according to differences in study design (Figure 3A), sample size (Figure 3B), mean age of the patients (Figure 4A), proportion of men (Figure 4B), dialysis vintage (Figure 5A), and STS treatment duration (Figure 5B, p for subgroup differences all > 0.05).

### Publication bias

**Figure 6.**
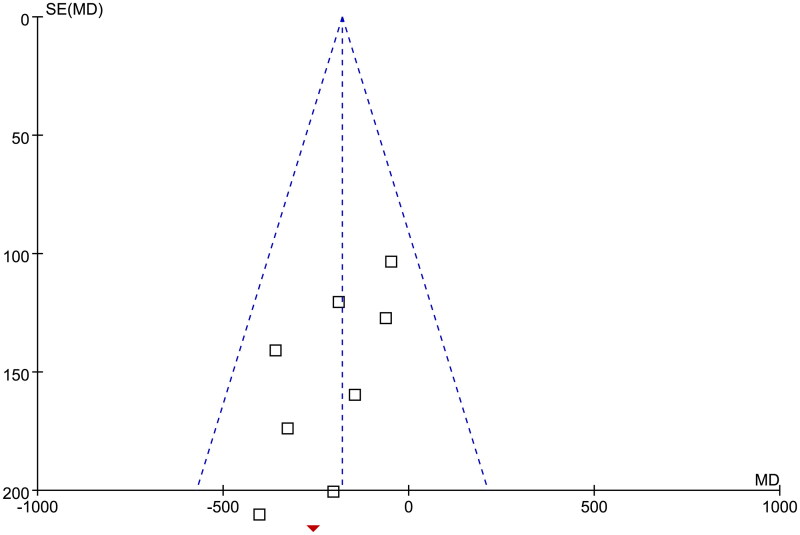
Funnel plots for the publication bias underlying the meta-analysis of the influence of intravenous STS on CAC scores in dialysis patients. Funnel plots of SE(MD) by MD were constructed for assessment of publication bias. Each open square denotes a study included in the meta-analysis. The dashed vertical line represents the overall effect of the meta-analysis.

The funnel plots for meta-analyses of the influence of STS on CAC scores in dialysis patients were symmetrical, suggesting a low risk of publication bias (Figure 6). Egger’s regression test also suggested a low risk of publication bias (*p* = 0.34).

## Discussion

In this systematic review and meta-analysis, we integrated the evidence from nine controlled clinical studies, and the results showed that, compared to the control group of usual care only, intravenous STS was associated with a significantly reduced CAC score in patients on dialysis. In addition, consistent results were obtained in the sensitivity analysis by excluding one study at a time and limiting it to patients on hemodialysis alone. Moreover, subgroup analyses showed that the results were not significantly influenced by multiple predefined study characteristics, such as study design, sample size, age, sex, dialysis vintage of the patients, and treatment duration of STS. Taken together, these results suggest that intravenous STS may be effective in attenuating CAC in dialysis patients.

To the best of our knowledge, few meta-analyses have examined the influence of STS treatment on CAC in dialysis patients. During the preparation of this manuscript, a meta-analysis of six studies of participants on maintenance hemodialysis showed that Intravenous STS may attenuate the progression of vascular calcification in hemodialysis patients. However, regarding the outcome of the CAC score, only three studies with 107 patients were available for the meta-analysis, which made the results less reliable. A few methodological strengths of the current meta-analysis should be noted compared to the previous meta-analysis. First, an extensive literature search was performed in five commonly used English and Chinese electronic databases, which retrieved nine controlled clinical studies based on the objective of the meta-analysis. In addition, both studies of patients on hemodialysis and peritoneal dialysis were included because previous studies suggested that there seemed to be no apparent differences in the risks of CAC progression in patients with these two dialysis modalities [9]. Moreover, the relatively large number of studies and patients included in this meta-analysis enabled us to perform multiple sensitivity and subgroup analyses to confirm the stability of the findings. For example, although NRCTs and RCTs were both included in the meta-analysis, a subgroup of RCTs only (seven studies) confirmed similar results. Although large-scale RCTs should also be performed to validate these findings, our meta-analysis indicates that intravenous STS for 3–6 months may effectively attenuate CAC progression in patients on dialysis.

In patients with CKD undergoing dialysis, CAC progression is not the only simple marker of coronary atherosclerosis. Previous studies have confirmed that CAC progression in dialysis patients is independently associated with an increased risk of all-cause mortality and MACEs [34,35], suggesting that attenuation of CAC progression may confer additional clinical benefits for these patients [36]. A recent study highlighted the importance of attenuation of CAC in dialysis patients by showing that despite the CAC score at baseline, CAC progression was also correlated with mortality in these patients [37]. Our meta-analysis showed that intravenous STS could significantly attenuate the progression of CAC in dialysis patients, which suggests that intravenous STS on the basis of usual care may be effective in improving survival and reducing MACEs in these patients. Well-designed long-term clinical trials should be considered in future studies.

The mechanisms underlying the influence of STS on CAC in patients undergoing dialysis remain unclear. Early experimental studies suggested that STS may attenuate vascular calcification by enhancing acid- and/or chelation-induced urinary calcium loss [38]. Subsequent results in models of adipocyte-induced arterial calcification showed that STS could inhibit this calcification, which was accompanied by a decrease in the secretion of leptin and vascular endothelial growth factor (VEGF) from adipocytes [15]. Moreover, studies in models of adenine-induced vascular calcification showed that STS was effective in reducing lipid peroxidation and elevating antioxidant enzymes, which led to attenuated vascular calcification, as well as renal and brain protection [16,39]. A recent study in patients undergoing maintenance hemodialysis suggested that STS can alleviate the progression of vascular calcification by changing the levels of calcification factors, which involved the increased levels of Matrix Gla protein and fetuin-A, and lowered levels of fibroblast growth factor-23 and osteoprotegerin [40]. Further studies are required to determine the key molecular mechanisms involved in this process.

This study has several limitations. First, the quality of the included studies was considered moderate. Although seven RCTs were included, all were open-label studies. The results of this meta-analysis should be confirmed in double-blind placebo-controlled RCTs. Second, the optimal dose and frequency of intravenous STS for patients on dialysis remain unknown and should be evaluated in the future. The dose of intravenous STS varied significantly among the included studies (5.6 g to 12.5 g), and the treatment durations for STS were relatively short among the included studies (3–6 months). Accordingly, we could not determine the optimal dose and frequency of intravenous STS for patients on dialysis based on limited data included. Specifically, since metabolic acidosis is highly prevalent in dialysis patients and a known side effect for intravenous administration of STS [41], although none of the included studies reported the incidence of metabolic acidosis during the follow-up duration of 3–6 months, it is also important to determine the optimal protocols of STS treatment regarding the long-term safety. Moreover, the treatment duration for STS in the included studies was 3–6 months. The long-term efficacy of STS on CAC, as well as the long-term safety of the treatment in these patients, should be observed in the future. In addition, in real-world clinical practice, for most patients, STS was administered for the treatment of calciphylaxis, and the improvement of CAC may be in fact a potential collateral benefit, which may introduce a possible selection bias into the presumed efficacy of STS. Also, a recent study from China showed that the risk factors for vascular calcification in Chinese patients may be different from those confirmed in patients from the Western countries [42]. Accordingly, it is important to validate the effect of STS on CAC in patients of different ethnic background. Furthermore, the protocol of the meta-analysis was not prospectively registered online. Finally, as mentioned earlier, it would be interesting to evaluate whether intravenous STS can improve the clinical outcomes of patients on dialysis.

## Conclusions

In summary, this systematic review and meta-analysis indicated that intravenous STS treatment for 3–6 months could significantly improve CAC in patients on dialysis. Large-scale clinical trials are needed to validate these findings and determine the optimal STS dose. Considering the important role of CAC progression as a determinant of MACEs in patients on dialysis, results of the meta-analysis may suggest that intravenous STS may be a potential treatment for improving cardiovascular prognosis of these patients. Studies with longer follow-up durations should also be considered to evaluate the influence of intravenous STS on cardiovascular events in dialysis patients and to evaluate the long-term safety of the treatment.

## Supplementary Material

Supplemental Material

Supplemental Material

## Data Availability

Data sharing is not applicable to this article, as no datasets were generated or analyzed during the current study.
