# Supplementary material for: Influence of sodium thiosulfate on coronary artery calcification of patients on dialysis: a meta-analysis
Source: Ren Fail. 2023 Sep 27;45(2):2254569. doi: 10.1080/0886022X.2023.2254569 (PMC10538455; doi:10.1080/0886022X.2023.2254569)
Supplement: Supplemental Material [file IRNF_A_2254569_SM7015.pdf]

Search terms for PubMed

("Thiosulfates"[MeSH Terms] OR "thiosulfates/pharmacology"[MeSH Terms] OR "sodium thiosulfate"[Supplementary Concept] OR "disodium salt"[Tiab] OR "sodium thiosulfate"[Tiab] OR "hyposulfite"[Tiab] OR "nsc 45624"[Tiab] OR "s hydril"[Tiab] OR "sodium hyposulfite"[Tiab] OR "sodium thiosulphate"[Tiab] OR "sodothiol"[Tiab] OR "sulfactol"[Tiab] OR "sulfothiorine"[Tiab] OR "thiosulfate sodium"[Tiab] OR "thiosulphate sodium"[Tiab]) AND ("Vascular Calcification"[MeSH Terms] OR "coronary artery"[Tiab] OR Calci*[Tiab]) AND ("dialysis"[MeSH Terms] OR "hemodialysis"[Tiab])

Search terms for Embase

('sodium thiosulfate'/exp OR 'sodium thiosulfate' OR 'sodium thiosulfate*' OR 'thiosulfate*' OR 'thiosulfuric acid' OR 'disodium salt' OR 'hyposulfite'/exp OR 'hyposulfite' OR 'nsc 45624'/exp OR 'nsc 45624' OR 's hydril'/exp OR 'shydril' OR 'sodium hyposulfite'/exp OR 'sodium hyposulfite' OR 'sodium thiosulphate'/exp OR 'sodium thiosulphate' OR 'sodothiol'/exp OR 'sodothiol' OR 'sulfactol'/exp OR 'sulfactol' OR 'sulfothiorine'/exp OR 'sulfothiorine' OR 'thiosulfate sodium'/exp OR 'thiosulfate sodium' OR 'thiosulphate sodium'/exp OR 'thiosulphate sodium') AND [embase]/lim AND ('cardiovascular calcification'/exp OR 'coronary artery'/exp OR 'calci*':ti,ab) AND ('dialysis'/exp OR 'hemodialysis'/exp) AND [embase]/lim

Search terms for Cochrane Library

Information Classification: General

([Thiosulfates][MeSH Terms] OR (Thiosulfates) OR (disodium salt) OR (sodium thiosulfate) OR (hyposulfite) OR (nsc 45624) OR (s hydril) OR (sodium hyposulfite) OR (sodium thiosulphate) OR (sodothiol) OR (sulfactol) OR (sulfothiorine) OR (thiosulfate sodium) OR (thiosulphate sodium)) AND ([Vascular Calcification][MeSH Terms] OR (Coronary artery) OR (Calci*)) AND ("dialysis"[MeSH Terms] OR "hemodialysis")

Search terms for CNKI

(主题=硫代硫酸钠) AND (主题=钙化 OR 主题=冠状动脉) AND (主题=透析 OR 主题=血透)

Search terms for Wanfang

主题:(硫代硫酸钠) and (主题:(钙化) or 主题:(冠状动脉)) and (主题:(透析) or 主题:(血透))

Information Classification: General
